# Lipid Peroxidation in Diabetic Kidney Disease: Mechanism and Natural Solution

**DOI:** 10.3390/ijms26199764

**Published:** 2025-10-07

**Authors:** Yuxin Dong, Yanqing Tong

**Affiliations:** College of Traditional Chinese Medicine, Changchun University of Chinese Medicine, Changchun 130000, China; 23102570143@stu.ccucm.edu.cn

**Keywords:** DKD, lipid metabolism disorders, lipid peroxidation, reactive oxygen species, natural products

## Abstract

Diabetic kidney disease (DKD), as one of the most serious microvascular complications of diabetes, is the main cause of end-stage renal disease in the world. Lipid peroxidation plays a crucial role in the development and progression of DKD. Under conditions of high glucose and insulin resistance, renal lipid metabolism disorders result in abnormal accumulation of polyunsaturated fatty acids (PUFAs), which undergo lipid peroxidation via free radical chain reactions to generate reactive aldehydes. These substances not only directly damage the cell structure but can also be used as signaling molecules that activate pathways related to inflammation, fibrosis, and ferroptosis, eventually leading to glomerular sclerosis and tubulointerstitial fibrosis. Natural products have shown considerable application prospects in the treatment of DKD due to their multi-functional properties, including anti-inflammatory, antioxidant, and lipid-metabolism-regulating effects. To elucidate this, we conducted a systematic review of the literature available in electronic databases (including PubMed, Web of Science, and Scopus, and Google Scholar) from January 2000 to May 2025. This study further discusses the therapeutic effect and mechanism of natural products targeting lipid peroxidation in DKD. The results indicate that natural products are promising anti-lipid peroxidation drugs. Further clinical trials will be necessary to verify the safety and effectiveness of these natural compounds in clinical applications, thereby laying the foundation for developing novel treatment strategies for DKD.

## 1. Introduction

Diabetic kidney disease (DKD) is one of the most serious and common microvascular complications of diabetes. According to epidemiological statistics, approximately 537 million people globally suffered from diabetes in 2021, and more than 40% of them subsequently developed DKD, which has become the main cause of end-stage renal disease (ESRD) [1]. While current treatment methods, including strict glycemic and blood pressure control, sodium-dependent glucose transporters 2 inhibitors (SGLT2is), and renin–angiotensin–aldosterone system inhibitors (RAASis), can delay the progression of DKD to some extent, many patients still progress irreversibly to renal failure and require renal replacement therapy to sustain their lives [2,3]. Therefore, it remains a critical challenge to explore the underlying molecular mechanisms of DKD and identify effective therapeutic targets.

Studies have shown that lipid peroxidation is a key pathogenic process in DKD [4,5]. Under physiological conditions, intracellular free fatty acids, which serve as a key energy source, are efficiently metabolized in mitochondria mainly through the *β*-oxidation pathway. However, the hyperglycemic environment and insulin resistance in diabetes disrupt the balance between fatty acid synthesis and oxidation, leading to lipid metabolism disorders [6]. Lipid metabolism disorders contribute to abnormal deposition of polyunsaturated fatty acids (PUFAs) in renal cells, further exacerbating oxidative stress. The consequent massive release of reactive oxygen species (ROS) reacts with accumulated PUFAs in renal cells, initiating a free radical chain reaction of lipid peroxidation that ultimately generates multiple highly reactive aldehyde compounds [7]. These reactive aldehydes not only directly disrupt the cell membrane structure and compromise functional integrity but also act as signaling molecules that trigger inflammatory responses, promote fibrotic processes, and induce cell death such as ferroptosis, ultimately aggravating the pathological progression of glomerulosclerosis and tubulointerstitial fibrosis.

In this complex pathological network, natural products exhibit unique therapeutic potential owing to their multi-targeted and multi-pathway characteristics. Studies have shown that various natural active ingredients (such as flavonoids, terpenoids and polyphenols) can effectively interrupt the vicious circle of lipid peroxidation by modulating the balance of fatty acid synthesis and oxidation, enhancing the antioxidant defense system and inhibiting inflammatory signal transduction, thereby demonstrating broad application prospects in DKD treatment [8]. In this paper, we summarize the molecular mechanism of lipid peroxidation in DKD, with a focus on its driving factors, signaling pathways and downstream effects, and discuss the current status of natural components targeting lipid peroxidation in the treatment of DKD, aiming to provide new insights for DKD treatment strategies.

## 2. Mechanisms of Lipid Peroxidation in DKD

### 2.1. Lipid Metabolism Disorder

**Figure 1:**
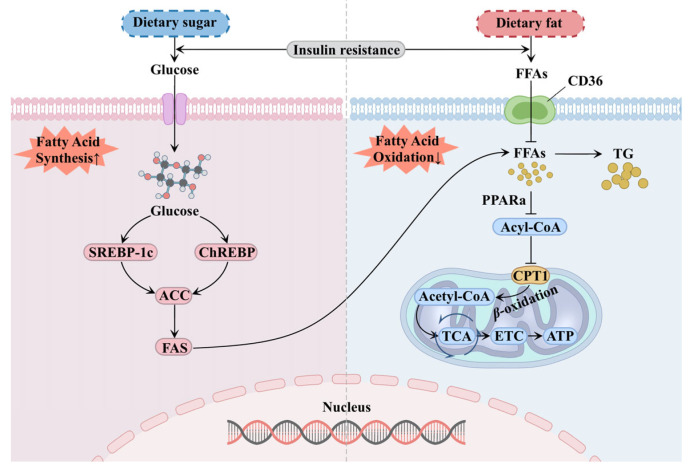
Lipid metabolism disorder in DKD. Hyperglycemia and insulin resistance in diabetes promote fatty acid synthesis and inhibit fatty acid oxidation, further esterifying to form triglycerides, leading to renal ectopic lipid deposition. Abbreviations: SREBP-1c, sterol regulatory element binding protein-1c; ChREBP, carbohydrate response element binding protein; ACC, acetyl-CoA carboxylase; FAS, fatty acid synthase; FFAs, free fatty acids; PPARa, peroxisome proliferator-activated receptor alpha; CPT1, carnitine palmitoyl transferase 1; TCA, tricarboxylic acid; ETC, electronic transfer chain; TG, triglycerides.

In the occurrence and development of DKD, lipid metabolism disorders play a critical role, with insulin resistance being recognized as the initiating factor [9]. Insulin exerts an anti-lipolysis function. However, in diabetic patients, impaired insulin sensitivity leads to enhanced lipolysis, resulting in the release of excessive free fatty acids (FFAs) into the circulatory system [10,11]. These surplus FFAs circulate to the kidney and are actively taken up into renal cells via upregulated scavenger receptors, such as cluster of differentiation 36 (CD36) [12]. Moreover, under the influence of persistent hyperglycemia and insulin resistance in DKD, fatty acid oxidation (FAO) is suppressed, while fatty acid synthesis is upregulated. Consequently, excess FFAs accumulate in the kidney in the form of triglycerides, which aggravates lipid metabolism disorders in DKD [7,13] (Figure 1).

Fatty acid oxidation (FAO) is a continuous biochemical process in which FFAs produce acetyl coenzyme A (Acetyl-CoA) and release ATP through a series of enzymatic reactions. Among them, fatty acid-*β*-oxidation represents the predominant form. FFAs are activated in the cytoplasm to form Acyl Coenzyme A (Acyl-CoA), which is then transported into mitochondria via carnitine palmitoyl transferase 1 (CPT1). Inside mitochondria, Acyl-CoA undergoes repeated *β*-oxidation to produce acetyl-CoA, which enters the tricarboxylic acid (TCA) cycle for complete oxidation, thereby generating energy through the mitochondrial electron transport chain (ETC) [14]. Mitochondria play an essential role throughout this process, particularly in highly energy-consuming renal tubular epithelial cells. FAO serves as the primary energy source sustaining the normal ion gradient, reabsorption function, and cellular viability of these cells [15]. Peroxisome proliferator-activated receptor alpha (PPARa) acts as the master regulator of FAO, governing the gene expression of a series of key enzymes in this pathway, while CPT1 functions as the principal carrier mediating fatty acid uptake into mitochondria [16]. However, renal biopsies from DKD patients have revealed downregulation of PPARa and its downstream target CPT1, accompanied by significant lipid deposition in renal tissues with an increase in intracellular lipid droplets [17]. Research on DKD animal models has confirmed that overexpression of CPT1 can down-regulate fibronectin expression, reduce inflammatory cell infiltration and mitochondrial damage, and improve renal function [18].

Fatty acid synthesis is a key metabolic pathway driving lipid deposition and is closely associated with hyperglycemia and insulin resistance in DKD. This process is primarily regulated by two key transcription factors: sterol regulatory element binding protein-1c (SREBP-1c) and carbohydrate response element binding protein (ChREBP) [19,20]. Under persistent high glucose conditions, both pathways are aberrantly activated and act synergistically to upregulate acetyl-CoA carboxylase (ACC) and fatty acid synthase (FAS), ultimately promoting fatty acid synthesis [21]. Studies have confirmed that the expression of lipogenic markers—SREBP-1c, ACC and FAS—is significantly up-regulated in DKD rats, and targeted therapy can mitigate the effect of hyperglycemia on renal lipogenesis [22,23].

Therefore, persistent hyperglycemia and insulin resistance in DKD can induce FFAs overload in the kidney, leading to subsequent esterification and triglyceride (TG) synthesis, and ultimately contributing to renal ectopic lipid deposition [24]. This ectopic lipid deposition impairs mitochondrial function and integrity, prompting excessive reactive oxygen species release, inducing oxidative stress and accelerating lipid peroxidation [25].

### 2.2. Oxidative Stress and Lipid Peroxidation

**Figure 2:**
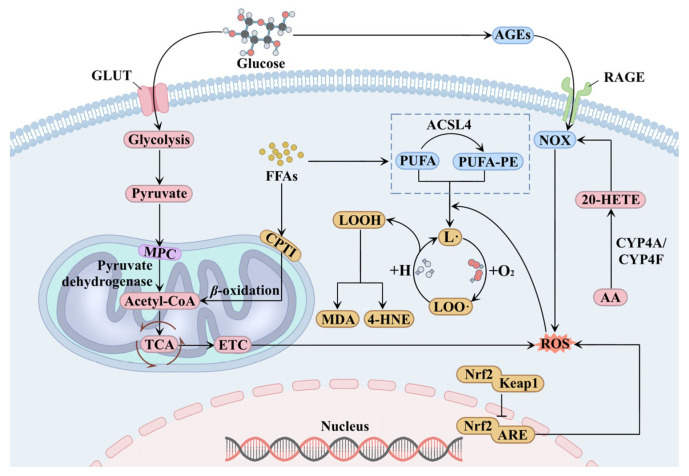
Oxidative stress and lipid peroxidation in DKD. Oxidative stress serves as the key driver of lipid peroxidation. Hyperglycemia and renal ectopic lipid deposition impair mitochondrial function. Furthermore, advanced glycation end products (AGEs) produced by plasma glucose bind to the receptor for AGE (RAGE), along with alterations in cytochrome P450 (CYP) enzyme activity, exacerbating oxidative stress and generating excessive reactive oxygen species (ROS). Concurrently, impaired nuclear factor erythroid 2-related factor 2 (Nrf2) function in DKD disrupts the antioxidant defense system. The massive ROS triggers a chain reaction of non-enzymatic lipid peroxidation, promoting the production of lipid peroxides. Abbreviations: GLUT, glucose transporter; MPC, mitochondrial pyruvate carrier; NOX, NADPH oxidase; Keap1, kelch-like ECH-associated protein 1; ARE, antioxidant response element; PUFA, polyunsaturated fatty acid; PUFA-PE, polyunsaturated fatty acid-phosphatidyl ethanolamine; ACSL4, Acyl-CoA synthetase long chain family member 4; AA, arachidonic acid; 20-HETE, 20-hydroxyeicosatetraenoic acid.

Under physiological conditions, fatty acid oxidation and fatty acid synthesis are maintained in a dynamic equilibrium. When energy supply is sufficient, fatty acids are synthesized within cells and subsequently esterified for storage. Conversely, when energy demand increases, fatty acids are oxidized to generate ATP [26]. However, in DKD, this lipid homeostasis is disrupted, leading to the accumulation of FFAs in the kidney. Hyperglycemia and renal lipid deposition further impair mitochondrial structure and function, promote oxidative stress, stimulate excessive ROS production, and trigger lipid peroxidation [25] (Figure 2).

Mitochondria serve as the primary site for cellular energy production. As an organ with high energy demand, the kidney is critically dependent on mitochondrial function [27]. However, studies have revealed that DKD is frequently associated with varying degrees of mitochondrial dysfunction and structural abnormalities, which may be closely linked to hyperglycemia and lipid metabolism disorders [28]. The persistent hyperglycemia in DKD upregulates glucose uptake via renal glucose transporter (GLUT), enhances glycolysis to generate pyruvate, and facilitates its entry into mitochondria through the mitochondrial pyruvate carrier (MPC) located on the inner mitochondrial membrane. Within the mitochondrial matrix, pyruvate is converted to acetyl coenzyme A (acetyl-CoA) by the pyruvate dehydrogenase complex [29]. Simultaneously, lipid metabolism disorders in DKD results in intracellular lipid accumulation, and a portion of FFAs enters mitochondria via CPT1 on the outer mitochondrial membrane to undergo *β*-oxidation, synergistically promoting the formation of acetyl-CoA. The acetyl-CoA derived from both glycolysis and fatty acid oxidation enters the TCA cycle, generating a substantial amount of energy carriers. This overload forces the ETC into a highly reduced, electron-saturated state, which favors the aberrant formation of ROS through the unintended transfer of electrons to molecular oxygen [30].

Advanced glycation end products (AGEs), resulting from hyperglycemia, also represent a major contributor to oxidative stress. AGEs are a class of heterogeneous molecules formed through the non-enzymatic glycation process, in which excess carbonyl groups of plasma glucose molecules react with amino groups of biomolecules such as lipids and proteins through nucleophilic addition [31]. In DKD, AGEs accumulate extensively in the kidney and bind to the receptor for AGE (RAGE). This binding triggers the recruitment and activation of NADPH oxidase (NOX), leading to the production of large amounts of ROS, which induces oxidative stress and exacerbates renal injury [32,33,34]. Furthermore, multiple studies have confirmed that inhibition of the AGE-RAGE signaling axis effectively suppresses NOX-mediated ROS production and alleviates oxidative renal damage, thereby further substantiating the pathogenic role of this pathway in DKD [35,36].

Beyond the classic oxidative stress pathways, alterations in cytochrome P450 (CYP) enzyme activity also serve as upstream drivers of oxidative stress in DKD. Under DKD conditions, the enhanced activity of renal CYP4A and CYP4F subfamilies shifts arachidonic acid (AA) metabolism toward the predominant production of 20-hydroxyeicosatetraenoic acid (20-HETE). As a key functional mediator, 20-HETE potently activates NOX, catalyzing ROS generation and aggravating lipid peroxidation damage [37,38].

Nuclear factor erythroid 2-related factor 2 (Nrf2) is a central regulator of cellular redox homeostasis. Under basal conditions, Nrf2 combines with its endogenous inhibitor kelch-like ECH-associated protein 1 (KeaP1) to form a complex, while in the oxidative stress state, Nrf2 undergoes nuclear translocation and binds with antioxidant response element (ARE), thereby activating the transcription of a battery of antioxidant genes [39,40]. Nevertheless, study has indicated that the function of Nrf2 may be inhibited in DKD, leading to a compromised antioxidant defense system that exacerbates renal oxidative injury [41].

Lipid peroxidation proceeds via both enzymatic and non-enzymatic pathways, with the non-enzymatic, free radical-mediated mechanism playing a predominant role in DKD progression [42,43]. In renal cells of DKD, abundant free PUFAs are esterified by acyl-CoA synthetase long chain family member 4 (ACSL4) to form polyunsaturated fatty acid-phosphatidyl ethanolamine (PUFA-PE). The initiation of non-enzymatic lipid peroxidation depends on reactions between ROS and these PUFAs or PUFA-PE. ROS extract hydrogen atoms from the carbon chain of PUFAs or PUFA-PEs, generating unstable lipid free radicals (L·). This rapidly reacts with oxygen to form lipid peroxide free radicals (LOO·), which can abstract hydrogen atoms from adjacent lipids to yield lipid hydroperoxides (LOOH) and new L·, thereby forming a self-sustaining chain reaction [44,45]. Additionally, LOOH can be further degraded to reactive secondary products such as malondialdehyde (MDA) and 4-hydroxy-nonenal (4-HNE). These reactive aldehydes exhibit high reactivity and are capable of mediating diverse physiological and pathological processes [46,47,48].

### 2.3. Lipid Peroxide-Mediated Renal Injury

**Figure 3:**
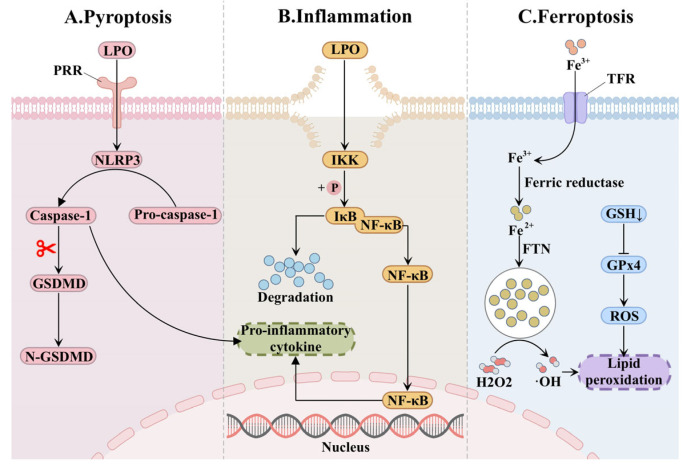
Lipid peroxide-mediated renal injury through (**A**) Pyroptosis, (**B**) Inflammation, and (**C**) Ferroptosis. Lipid peroxide (LPO) is a key mediator leading to renal damage. It exacerbates kidney injury by disrupting cell membrane structure, modifying protein function, and acting as a signaling molecule. LPO can activate the nuclear factor kappa-B (NF-κB) and NOD-like receptor thermal protein domain associated protein 3 (NLRP3) inflammasome pathways, triggering caspase-1-mediated cleavage of GSDMD and promoting inflammatory responses and pyroptosis. Meanwhile, LPO inhibits the activity of glutathione peroxidase 4 (GPx4), weakening the antioxidant defense system. It also synergizes with intracellular iron overload to drive ferroptosis, a specific form of programmed cell death, thereby playing a central role in the progression of DKD. Abbreviations: PRR, pattern recognition receptors; GSDMD, gasdermin D; IKK, inhibitor of kappa B kinase; IκB, inhibitor of NF-κB; TFR, transferrin receptor; FTN, ferritin; GSH, glutathione.

Lipid peroxides (LPOs) generated through lipid peroxidation serve as both direct effector substances and signaling molecules of cellular damage. The cross-linking of LPO with phosphatidylcholine compromises membrane fluidity and selective permeability, thereby directly impairing normal cellular physiological functions [46,49]. In addition, reactive aldehydes derived from LPO, such as MDA and 4-HNE, exhibit strong electrophilic properties, enabling them to react readily with sulfhydryl or amino groups in proteins. This interaction leads to the formation of covalent adducts and subsequent protein dysfunction [50]. Therefore, LPOs also function as bioactive signaling molecules that modulate multiple pathways implicated in inflammation, oxidative stress and cell death in kidney diseases [51,52] (Figure 3).

Nuclear factor kappa-B (NF-κB) is an inducible transcription factor that directly regulates the expression of inflammatory mediators and plays a key role in inflammatory and immune responses in kidney diseases [53,54]. The activation of this signaling pathway primarily relies on phosphorylation of the inhibitor of NF-κB (IκB) mediated by the inhibitor of kappa B kinase (IKK) complex. Notably, LPO produced by lipid peroxidation can covalently modify IKK, forming adducts that promote IκB phosphorylation and subsequent nuclear translocation of NF-κB, thereby initiating transcription of proinflammatory cytokines and chemokines, and amplifying inflammatory injury [55,56]. Simultaneously, protein adducts derived from lipid peroxidation can act as damage associated molecular patterns (DAMPS) that bine to pattern recognition receptors (PRR) and activate NOD-like receptor thermal protein domain associated protein 3 (NLRP3) inflammasome [57]. Upon abnormal activation of the NLRP3 inflammasome, pro-caspase-1 is cleaved into caspase-1, which in turn promotes the release of the potent pro-inflammatory cytokines IL-1β and IL-18 [58,59,60]. Additionally, caspase-1 cleaves gasdermin D (GSDMD), generating N-terminal fragment of GSDMD (N-GSDMD) that forms membrane pores, and induces pyroptosis [61]. Consistent with these mechanisms, studies have confirmed that high glucose activates the NF-κB signaling pathway, upregulates the expression of IL-6, and contributes to glomerulosclerosis in DKD [62]. In high glucose-cultured glomerular mesangial cells, the phosphorylation level of IκBα is enhanced, further activating NF-κB signaling pathway, exacerbating inflammation, and promoting mesangial cell proliferation [63].

The abundant free radicals generated during the lipid peroxidation chain reaction can directly damage proteins, DNA and lipids, disrupt cellular redox homeostasis, and aggravate oxidative stress. Meanwhile, reactive aldehydes derived from unstable lipid peroxides covalently modify mitochondrial respiratory chain complexes, leading to ETC dysfunction and promoting mitochondrial ROS production [64]. Under physiological conditions, glutathione peroxidase (GPx) serves as a critical endogenous antioxidant enzyme that eliminates lipid hydroperoxides and terminates lipid peroxidation cascades [65]. However, studies have revealed that reactive aldehydes can also modify the glutathione (GSH) binding site, thereby inhibiting GPx activity, compromising the cellular antioxidant defense system, and contributing to the progression of oxidative stress [66,67].

Interestingly, the inhibition of GPx by reactive aldehydes may synergistically enhance ferroptosis in DKD. In the cellular iron cycle, Fe^3+^ bound to transferrin enters renal cells via transferrin receptor (TFR), is then reduced to Fe^2+^ by ferric reductases and stored within ferritin (FTN) to maintain cellular iron homeostasis [68]. However, in DKD, this homeostasis is disrupted. Excess labile iron promotes the Fenton reaction, generating highly reactive free radicals that initiate lipid peroxidation and trigger a unique form of regulated cell death known as ferroptosis, which is characterized by iron-dependent lipid peroxidation [69,70]. Studies in DKD animal models have demonstrated suppressed expression of GPX4 and GSH, accompanied by upregulation of TFR1, elevated levels of iron and MDA, collectively substantiating the involvement of ferroptosis in renal injury [71].

## 3. Natural Intervention Strategies for Lipid Peroxidation in Diabetic Kidney Disease

In the therapeutic strategy for DKD, natural products demonstrate significant potential. Studies have indicated that natural products can effectively counter lipid metabolism disorders in DKD, alleviate renal ectopic lipid deposition, mitigate oxidative stress status, and enhance antioxidant defenses, thereby suppressing lipid peroxidation. Furthermore, aberrant activation of lipid peroxidation exacerbates the progression of DKD by promoting inflammation, oxidative stress, ferroptosis, and other processes, while natural products represent a promising strategy to ameliorate this pathogenic cycle (Table 1).

### 3.1. Cutting Off the Substrate Supply: Restoring Lipid Metabolic Homeostasis

Resveratrol, a natural phytoalexin obtained from *Reynoutria japonica* Houtt (Polygonaceae), exhibits potent bioactivity. As a well-established Sirtuin 1 (SIRT1) agonist, it confers broad protection against lipid metabolism disorders [72,73]. Mechanistically, resveratrol activates SIRT1, suppressing lipogenic transcription factors SREBP-1 and ChREBP and significantly ameliorating renal ectopic lipid deposition [74]. In diabetic animal models, it also activates the SIRT1/PGC1 axis, upregulating PPARa and CPT1 to promote fatty acid-*β*-oxidation and ameliorate renal injury [75]. Notably, resveratrol suppresses the AGE/RAGE axis under high glucose conditions, thereby alleviating renal oxidative stress, inhibiting lipid peroxidation, significantly reducing 4-HNE levels, and attenuating DKD progression [76].

Curcumin, a natural polyphenol extracted from the rhizome of *Curcuma longa* L. (Zingiberaceae), exhibits hypolipidemic, anti-inflammatory, and antioxidant properties with therapeutic potential for endocrine and immune-related diseases [77]. It enhances adenosine 5′-monophosphate (AMP)-activated protein kinase (AMPK) phosphorylation, leading to downregulation of SREBP-1 and phosphorylation-mediated inactivation of ACC, thereby inhibiting fatty acid synthesis, reducing renal lipid deposition, and ameliorating glomerular basement membrane thickening in DKD [78].

Apigenin, a naturally abundant flavonoid in *Apium graveolens* L. (Apiaceae), improves insulin resistance and regulates glucose and lipid metabolism [79]. It suppresses SREBP-1c and SREBP-2 expression, markedly down-regulating FAS activity and attenuating ectopic lipid deposition [80]. Furthermore, studies have revealed that apigenin downregulates GLUT1 expression, curbs transmembrane glucose uptake, lowers serum TG and total cholesterol, collectively improving both insulin sensitivity and lipid homeostasis [81].

Soy isoflavones, polyphenolic compounds extracted from the seeds of *Glycine max* (L.) Merr (Fabaceae), demonstrate promise in preventing and managing of metabolic diseases. Studies have revealed that soy isoflavones activate PPARa via enhanced ligand binding, promoting transcription of target genes and enhancing fatty acid oxidation, thereby countering insulin resistance-induced lipid metabolism disorders [82]. In diabetic rats, soy isoflavones suppress renal SREBP-1c expression, inhibit fatty acid synthesis, and ameliorate glomerulosclerosis [83].

Anthocyanin, a potent natural antioxidant abundant in berries such as *Vaccinium uliginosum* L. (Ericaceae), exhibits strong free radical scavenging capacity and antioxidant activity. Additionally, anthocyanin can modulate lipid-metabolizing enzymes, thereby ameliorating lipid metabolism disorders and inhibiting lipid peroxidation [84]. Studies have found that anthocyanin regulates glucose and lipid metabolism primarily through AMPK activation, which suppresses the expression of key fatty acid synthesis enzymes SREBP-1c and ACC, inhibits gluconeogenesis and improves insulin sensitivity, ultimately ameliorating glucose and lipid metabolism disorders [85].

Myricetin, a major active component extracted from *Morella rubra* Lour (Myricaceae), also demonstrates lipid metabolism regulatory effects. It downregulates SREBP-1c and SREBP-2 while upregulating PPARa, exerting significant protective effects against diabetes-induced lipid metabolism disorders. Concurrently, myricetin suppresses the overexpression of transforming growth factor-β (TGF-β), thereby ameliorating glomerulosclerosis and interstitial fibrosis in DKD [86].

### 3.2. Reducing Triggers: Reinforcing the Antioxidant Barrier

Hesperidin, a flavonoid abundant in the peel of *Citrus reticulata* Blanco (Rutaceae), possesses potent free radical-scavenging capacity and enhances the expression of Nrf2 [87]. Research has shown that hesperidin augments Nrf2/ARE signaling transduction, promotes glyoxalase 1 overexpression, and subsequently suppresses AGE/RAGE activation, thereby ameliorating oxidative stress-induced renal pathology in DKD [88]. Furthermore, hesperidin significantly downregulates renal NF-κB and NLRP3 expression, reduces pro-inflammatory cytokines IL-6 and IL-1β, and mitigates renal injury driven by diabetic hyperglycemia and hyperlipidemia [89].

Quercetin, a flavonol widely present in various herbal plants, protects against tissue damage through its potent antioxidant capacity. In diabetic kidney disease, ROS derived primarily from mitochondrial and NOX activities contribute to oxidative stress, while quercetin acts as an effective ROS scavenger to maintain redox homeostasis [90]. It also activates Nrf2 signaling and alleviates high glucose-induced overexpression of TFR1 in renal tubular epithelial cells and upregulates GPx4 to ameliorate intracellular iron overload and lipid peroxidation [91]. Moreover, studies have demonstrated that quercetin significantly restores the activities of antioxidant enzymes—including catalase (CAT), superoxide dismutase (SOD), and glutathione S-transferase (GST)—under diabetic conditions. By enhancing antioxidant capacity, it inhibits lipid peroxidation, reduces levels of MDA, and ameliorates renal fibrosis, thus exerting renal protection [92].

Astragaloside IV, a triterpenoid extracted from the dried roots of *Astragalus membranaceus* (Fisch.) Bunge (Fabaceae), binds to Keap1 and promotes Nrf2 release and nuclear translocation. Within the nucleus, it binds to the ARE, synergistically upregulating multiple antioxidant enzymes. Additionally, it helps maintain mitochondrial homeostasis, restores the activity of mitochondrial respiratory chain complexes, and reduces the release of ROS [93]. Studies have found that astragaloside IV effectively ameliorates palmitate-induced oxidative stress damage in glomerular mesangial cells by downregulating CD36 expression, reducing active uptake of free fatty acids, suppressing the NOX subunit p22phox, and inducing NOX degradation, thereby alleviating lipid deposition and curbing ROS production [94,95]. Moreover, astragaloside IV significantly inhibits NLRP3 inflammasome activation, reduces IL-1β, TNF-α, and MCP-1 levels, and improves renal function and podocyte injury in DKD [96].

Sinapic acid, a phenolic compound found in various plants such as fruits, vegetables, and herbs, functions as a natural free radical scavenger due to its unique molecular structure. Its phenolic hydroxyl groups donate hydrogen atoms to neutralize free radicals, forming stable intermediates that terminate radical chain reactions and inhibit lipid peroxidation [97]. In DKD rats, the administration of sinapic acid activates Nrf2, upregulates SOD and GPx4, thereby significantly suppressing lipid peroxidation. Concurrently, it downregulates NF-κB signaling, inhibiting the release of IL-6 and TNF-α [98]. Furthermore, sinapic acid activates PPAR and CPT1, suppresses SREBP-1, ACC, and FAS expression, ameliorates lipid metabolism disorders, and enhances total antioxidant capacity, thereby mitigating oxidative stress [99].

Chlorogenic acid, a major phenolic compound found in *Lonicera japonica* Thunb. (Caprifoliaceae), also exhibits remarkable antioxidant capacity. Studies have reported that the protective effect of chlorogenic acid against DKD involves the interaction between the Nrf2/Heme Oxygenase-1 (HO-1) and NF-κB pathways. Upregulated Nrf2 activates HO-1, enhancing antioxidant enzyme expression, while HO-1 downregulates NF-κB and inhibits the release of pro-inflammatory cytokines, significantly ameliorating diabetic kidney injury [100]. Meanwhile, chlorogenic acid can also modulate the NLRP3 inflammasome via Nrf2, exerting anti-inflammatory effects [101].

Proanthocyanidin, obtained from the seeds of *Vitis vinifera* L. (Vitaceae), serves as an effective free radical scavenger with potent antioxidant activity. Studies have reported that proanthocyanidin activates the Keap1/Nrf2 signaling pathway, upregulates GSH and SOD, and mitigates lipid peroxidation-induced toxicity in DKD [102]. Furthermore, proanthocyanidin inhibits AGE formation. The phenolic hydroxyl groups of proanthocyanidin can trap the carbonyl groups of dicarbonyl compounds, which are precursors to AGEs, forming stable adducts and thereby blocking AGE generation. This action suppresses NOX activation via the AGE/RAGE axis, contributing to oxidative stress amelioration [103].

Geniposide, a natural bioactive compound isolated from *Gardenia jasminoides* J. Ellis (Rubiaceae), exhibits multiple pharmacological effects including anti-inflammatory, antioxidant, antidiabetic, and neuroprotective properties. Studies have demonstrated that geniposide activates protein kinase A (PKA) and enhances AMPK phosphorylation, thereby promoting autophagy and subsequently inhibiting oxidative stress [104]. Additionally, phosphorylated AMPK further activates SIRT1 and suppresses NF-κB, reducing inflammatory cell infiltration and glomerular basement membrane thickening [105].

Lycopene, a potent antioxidant obtained from *Solanum lycopersicum* L. (Solanaceae), effectively inhibits lipid peroxidation. It upregulates Nrf2, enhances renal antioxidant capacity, suppresses NF-κB signaling, lowers IL-1β and TNF-α levels, and improves renal inflammation and oxidative stress damage [106]. Concurrently, lycopene also reduces renal AGEs, upregulates SOD and CAT, promotes GPx expression, effectively scavenges free radicals, and alleviates oxidative stress, thereby inhibiting lipid peroxidation in DKD [107].

Coumarin derivatives extracted from the seeds of *Dipteryx odorata* (Aubl.) Willd. (Fabaceae) exert multi-target therapeutic effects by alleviating oxidative stress and fibrosis. They activate the Nrf2 signaling pathway to enhance antioxidant capacity and effectively scavenge ROS, and they also function as natural SIRT3 activators, promoting the deacetylation of forkhead box O3A (FOXO3a) to enhance manganese superoxide dismutase (MnSOD) activity, thereby establishing a dual antioxidant defense. Concurrently, coumarin derivatives inhibit Smad2/3 phosphorylation, reducing the secretion of fibronectin and delaying renal fibrosis. Furthermore, they downregulate renal SREBP expression, suppress fatty acid synthesis, and ameliorate renal ectopic lipid deposition [108,109].

### 3.3. Ameliorating Renal Damage: Delaying the Progression of Kidney Pathology

Silymarin, an extract obtained from the dried fruits and seeds of *Silybum marianum* (L.) Gaertn (Asteraceae), exhibits anti-inflammatory, antioxidant, and anti-fibrotic properties. Studies have found that silymarin ameliorates renal fibrosis in DKD, potentially through inhibition of the TGF-β/Smad signaling pathway [110]. TGF-β, a major driver of fibrosis, is significantly upregulated in DKD, leading to Smad protein activation, nuclear translocation, and subsequent expression of fibrotic genes [111]. Additionally, silymarin upregulates antioxidant enzymes including CAT and GPx4, thereby alleviating lipid peroxidation in DKD [112].

α-Actinin-4 (ACTN4), an actin-binding protein, is critical for maintaining the structural and functional integrity of glomerular podocytes [113]. In DKD, however, hypermethylation of ACTN4 occurs, compromising the glomerular filtration barrier and promoting scar formation [114]. Epigallocatechin gallate, a compound isolated from *Camellia sinensis* (L.) Kuntze (Theaceae), exhibits natural demethylating activity and demonstrates therapeutic potential in DKD. Studies have confirmed that Epigallocatechin gallate inhibits DNA methyltransferases, reverses ACTN4 hypermethylation, and suppresses the expression of NF-κB and IκBα. This leads to reduced levels of the fibrosis marker α-smooth muscle actin (α-SMA) and inflammatory cytokines, thereby ameliorating renal damage [115]. Additionally, Epigallocatechin gallate inhibits the overexpression of NLRP3 and blocks the release of IL-1β, thereby attenuating DKD-associated inflammatory injury [116].

Isoliquiritigenin, a bioactive compound extracted from the roots of *Glycyrrhiza uralensis* Fisch (Fabaceae), demonstrates potent free radical scavenging capacity, ameliorates oxidative stress, and inhibits lipid peroxidation. Additionally, it suppresses NLRP3 and NF-κB signaling pathways, exerting anti-inflammatory and antioxidant effects that alleviate renal damage in DKD [89]. Furthermore, 4-HNE, an active mediator linking lipid peroxidation and inflammatory responses, has been demonstrated in other disease models to modify the granulocyte-macrophage colony-stimulating factor (GM-CSF) receptor, leading to sustained activation of the downstream janus kinase (JAK)/signal transducer and activator of transcription (STAT) signaling pathway and amplified inflammatory responses [117,118]. The role of JAK/STAT signaling in DKD-related inflammatory damage is well-established [119]. Studies have found that isoliquiritigenin ameliorates renal inflammation and fibrosis partly through inhibition of the JAK/STAT signaling pathway. In DKD, upregulated JAK promotes the phosphorylation and nuclear translocation of STAT, regulating pro-inflammatory cytokines and fibrotic markers. Isoliquiritigenin treatment significantly inhibits the JAK2/STAT3 signaling pathway, downregulates TGF-β, fibronectin, and collagen expression, and improves renal pathological damage in DKD [120].

Andrographolide, the primary active constituent obtained from *Andrographis paniculata* (Burm. f.) Nees (Acanthaceae), is known as a “natural antibiotic” for its notable anti-inflammatory and antiviral properties. Studies have revealed that andrographolide ameliorates renal pathology in DKD by suppressing the STAT3-mediated phosphoinositide 3-kinase (PI3K)/protein kinase B (Akt) signaling pathway. Aberrant activation of PI3K promotes DKD progression by recruiting and phosphorylating Akt, thereby promoting inflammation and inducing epithelial–mesenchymal transition (EMT) in renal tubular epithelial cells. Andrographolide effectively reverses this process [121]. Moreover, andrographolide induced Akt inhibition reduces NF-κB transcriptional activity, blocks pro-inflammatory cytokines release, and mitigates inflammatory injury in DKD [122].

Berberine, an alkaloid isolated from the roots of *Coptis chinensis* Franch (Ranunculaceae), exhibits hypoglycemic and lipid-lowering, and insulin-sensitizing effects, demonstrating considerable potential for treating diabetes and its complications [123]. Furthermore, studies have shown that berberine also inhibits ferroptosis by upregulating GPx4 and Nrf2, thereby effectively inhibiting ferroptosis [124].

Ginkgolide B, primarily extracted from the dried leaves of *Ginkgo biloba* L. (Ginkgoaceae), is well-known for its specific platelet-activating factor receptor antagonist with recently demonstrated renal protective effects. In vitro, ginkgolide B inhibits TFR1, enhances FTH1 expression, reduces overloaded intracellular free iron levels, and restores renal iron homeostasis. It also suppresses ubiquitination-mediated degradation of GPx4, thereby improving antioxidant capacity and inhibiting oxidative stress and ferroptosis in DKD [125]. Furthermore, ginkgolide B activates PPARa to promote fatty acid oxidation and upregulates the Nrf2-mediated antioxidant system, thus ameliorating lipid metabolism disorders [126].

Tanshinone IIA, a diterpenoid quinone found in the roots of *Salvia miltiorrhiza* Bunge (Lamiaceae), is used in treating cardiovascular and cerebrovascular diseases. Studies have shown that tanshinone IIA exhibits renal protective effects, including anti-inflammatory, antioxidant, and anti-fibrotic activities. In DKD models, tanshinone IIA inhibits the activation of NLRP3, blocking caspase-1-mediated release of pro-inflammatory cytokines and cleavage of GSDMD, thereby suppressing pyroptosis [127]. Furthermore, it also downregulates ACSL4 expression, attenuates high glucose-induced ferroptosis, upregulates GSH to scavenge free radicals, and collectively ameliorates renal pathological damage in DKD [128].

**Table 1:** Natural products regulating lipid peroxidation in DKD.

Natural Product	Source	Application Model	Mechanism	Refs.
Resveratrol	*Reynoutria japonica* Houtt	HFD induced C57BL/6J mice C57BL/KsJ db/db mice HG induced NMS2 cells STZ injected male Wistar rats	Activate SIRT1/PGC1, upregulate PPARa/CPT1, suppress SREBP-1 and ChREBP, suppress AGE/RAGE, reduce 4-HNE	[74,75,76]
Curcumin	*Curcuma longa* L.	OLETF rats	Enhance AMPK phosphorylation, downregulate SREBP-1 and promote ACC phosphorylation	[78]
Apigenin	*Apium graveolens* L.	HFD induced male C57BL/6J mice HF induced SD rats	Suppress SREBP-1c and SREBP-2, downregulate FAS and GLUT1	[80,81]
Soy isoflavones	*Glycine max* (L.) Merr	obese Zucker rats HFD + STZ injected male SD rats	Active PPARa and suppress SREBP-1c	[82,83]
Anthocyanin	*Vaccinium uliginosum* L.	HFSD + STZ injected male C57BL/6J mice	Scavenge free radicals, activate AMPK, suppress SREBP-1c and ACC	[85]
Myricetin	*Morella rubra* Lour	STZ-Cd induced male albino Wistar rats	Downregulate SREBP-1c and SREBP-2, upregulate PPARa, suppress TGF-β	[86]
Hesperidin	*Citrus reticulata* Blanco	STZ injected male SD rats HFD + STZ injected Wistar rats	Scavenge free radicals, activate Nrf2/ARE signaling pathway, promote Glo-1 and suppress AGE/RAGE, downregulate NF-κB and NLRP3, reduces IL-6 and IL-1β	[88,89]
Quercetin	Various herbal plants	HG induced HK-2 cells Male C57BL/KsJ db/db mice	Scavenge ROS, activate Nrf2, downregulate TFR1 and upregulate GPx4, enhance CAT, SOD and GST	[91]
Astragaloside IV	*Astragalus membranaceus* (Fisch.) Bunge	STZ injected male C57BL/6J rats HG induced MPC cells HFD + STZ injected Sprague Dawley rats PA induced HMC cells BKS-db/db mice HG induced MPC cells	Promote Nrf2, downregulate CD36, suppress NOX, inhibit NLRP3 inflammasome, reduce IL-1β, TNF-α, and MCP-1	[93,94,96]
Sinapic acid	Various herbal plants	STZ injected male Wistar rats HFD induced male Syrian hamsters	Scavenge ROS, activate Nrf2, upregulate SOD and GPx4, downregulate NF-κB signaling pathway, promote PPAR and CPT1, suppress SREBP-1, ACC, and FAS	[98,99]
Chlorogenic acid	*Lonicera japonica* Thunb.	STZ injected HBZY-1 cells HFD + STZ injected Wistar rats HG induced HK-2 cells	Upregulate Nrf2/HO-1, downregulate NF-κB and NLRP3 inflammasome signaling pathway	[100,101]
Proanthocyanidin	*Vitis vinifera* L.	Cd + HFSD induced male KM mice	Scavenge ROS, activate Keap1/Nrf2 signaling pathway, upregulate GSH and SOD, suppress NOX via the AGE/RAGE axis	[102]
Geniposide	*Gardenia jasminoides* J. Ellis	HFD + UNx + STZ injected C57BL/6 male mice HFD + STZ injected C57BL/6 mice	Activate PKA, enhance AMPK and autophagy, activate SIRT1 and suppress NF-κB	[104,105]
Lycopene	*Solanum lycopersicum* L.	HFD induced male Wistar rats STZ injected male Wistar rats	upregulate Nrf2, suppress NF-κB, reduce IL-1β and TNF-α, suppress AGEs, upregulate SOD and CAT, promotes GPx	[106,107]
Coumarin derivatives	*Dipteryx odorata* (Aubl.) Willd.	STZ injected male Wistar rats HG induced HBZY-1 cells HFD + STZ injected male C57BL/6 mice AGEs induced HK2 cells	Activate Nrf2, scavenge ROS, activate SIRT3, promote FOXO3a, enhance MnSOD, inhibit Smad2/3, reduce fibronectin, downregulate SREBP	[108,109]
Silymarin	*Silybum marianum* (L.) Gaertn	HG induced MPC5 cells STZ injected male SD rats	Inhibit TGF-β/Smad signaling pathway, upregulate CAT and GPx4	[110,112]
Epigallocatechin gallate	*Camellia sinensis* (L.) Kuntze	HG induced HPC cells Male C57BL/KsJ db/db mice	Inhibit DNA methyltransferase, reverse ACTN4 hypermethylation, suppress NF-κB and IκBα, reduce α-SMA and inhibit NLRP3	[115,116]
Isoliquiritigenin	*Glycyrrhiza uralensis* Fisch	HFD + STZ injected Wistar rats HG induced HK-2 cells HFD + STZ injected male SD rats	Scavenge free radicals, suppress NLRP3, NF-κB and JAK/STAT signaling pathways, downregulate TGF-β, fibronectin, and collagen	[89,120]
Andrographolide	*Andrographis paniculata* (Burm. f.) Nees	HFD + STZ induced SD rats AGEs induced MPC5 cells HFD + STZ injected C57BL/6 mice	Suppress STAT3/PI3K/Akt signaling pathway, inhibit NF-κB	[121,122]
Berberine	*Coptis chinensis* Franch	HFD induced C57BL/6J mice and AopE−/− mice	Upregulate GPx4 and Nrf2	[124]
Ginkgolide B	*Ginkgo biloba* L.	Male C57BL/KsJ db/db mice PA-G induced MPC5 cells HFD induced SD rats	Inhibit TFR1, enhance FTH1, reduce intracellular free iron, suppress the ubiquitination of GPx4, activate PPARa and upregulate Nrf2	[125,126]
Tanshinone IIA	*Salvia miltiorrhiza* Bunge	db/db mice HG induced HRGEC cells HG induced MPC5 cells	Inhibit NLRP3/caspase-1/GSDMD mediated pyroptosis, downregulate ACSL4, upregulate GSH and scavenge free radicals	[127,128]

## 4. Conclusions

Diabetic kidney disease (DKD) is a complex pathological process characterized by the interplay of chronic hyperglycemia and lipid metabolism disorders, involving multiple interconnected mechanisms. In recent years, lipid metabolism disorders, particularly ectopic lipid deposition in the renal tissue and the resulting lipid peroxidation, have emerged as a core factor promoting the progression of DKD. This process accelerates glomerulosclerosis and tubulointerstitial fibrosis by disrupting cellular structures, amplifying inflammatory responses, exacerbating oxidative stress, and inducing programmed cell death, ultimately leading to irreversible renal injury. Therefore, targeting lipid peroxidation and its associated injury network represents a highly promising therapeutic strategy for DKD.

Although current studies have made significant progress, the pathogenic network of DKD requires further systematic elucidation. For instance, 4-HNE is not merely a terminal product of lipid peroxidation but also functions as a highly reactive signaling molecule that actively contributes to disease progression. However, its specific mechanisms of action in DKD still need further exploration. Future studies should prioritize elucidating the molecular regulatory network of lipid peroxidation in DKD, systematically identifying the specific targets, signaling pathways, and interactions of 4-HNE with other pathological processes. A deeper understanding of these mechanisms will not only refine the pathological framework of DKD but also provide novel insights and targets for developing natural medicines that intervene in lipid peroxidation and its downstream effects.

Traditional Chinese herbal medicine possesses a rich historical foundation in disease treatment. However, the poorly defined active constituents have often limited its application and promotion in modern medicine. With advances in extraction and isolation technologies, our understanding of bioactive compounds obtained from natural plants has considerably improved. Natural compounds extracted from medicinal herbs have demonstrated remarkable efficacy in mitigating lipid peroxidation in DKD. These components ameliorate renal pathology through core signaling pathways such as FAO, FAS, Nrf2, GPx4, and NF-κB, which represent key regulators of lipid peroxidation in DKD. Preclinical studies have preliminarily confirmed the potential of natural compounds in alleviating lipid peroxidation in DKD. These compounds exhibit diverse mechanisms of action and represent promising prospects for novel therapeutic strategies, given their capacity to modulate multiple targets and pathways in a synergistic manner.

It is noteworthy that the multi-target and multi-pathway characteristics of natural products, while offering comprehensive therapeutic advantages, also entail potential off-target risks, complicating their clinical application. Moreover, most current evidence comes from animal or cell-based studies, and clinical translation still faces multiple challenges. The complexity of human physiology necessitates further validation of critical issues such as dose–response relationships, long-term safety, and potential drug interactions. Additionally, standardized extraction processes and rigorous quality control are essential prerequisites for clinical application. Nevertheless, the considerable potential of natural products in mitigating lipid peroxidation in DKD should not be overlooked.

Future studies should focus on further elucidating the molecular mechanisms underlying lipid peroxidation in DKD to identify novel therapeutic targets. Moreover, natural products often suffer from limitations such as low oral bioavailability and rapid systemic clearance, which hinder their therapeutic efficacy. Therefore, research efforts should be directed toward developing advanced drug delivery systems—such as nanoparticles, liposomes, or kidney-targeting peptide carriers—to improve bioavailability and renal targeting. At the same time, it is crucial to conduct large-scale, multicenter, randomized controlled clinical trials to systematically evaluate the efficacy, long-term safety, and potential side effects of natural products in DKD patients, thereby providing a robust evidence base for their clinical translation.

## Data Availability

No new data were created or analyzed in this study. Data sharing is not applicable to this article.

## Abbreviations

The following abbreviations are used in this manuscript:

4-HNE	4-Hydroxy-nonenal
ACC	Acetyl-CoA carboxylase
ACSL4	Acyl-CoA synthetase long chain family member 4
AMPK	Adenosine 5′-monophosphate (AMP)-activated protein kinase
AGE	Advanced glycation end product
ARE	Antioxidant response element
ChREBP	Carbohydrate response element binding protein
CPT1	Carnitine palmitoyl transferase 1
CAT	Catalase
CD36	Cluster of differentiation 36
DKD	Diabetic kidney disease
ETC	Electron transport chain
ESRD	End-stage renal disease
EMT	Epithelial–mesenchymal transition
FAO	Fatty acid oxidation
FAS	Fatty acid synthase
FTN	Ferritin
GSDMD	Gasdermin D
GLUT	Glucose transporter
GSH	Glutathione
GPx	Glutathione peroxidase
GST	Glutathione S-transferase
HO-1	Heme oxygenase-1
IKK	Inhibitor of kappa B kinase
IκB	Inhibitor of NF-κB
JAK	Janus kinase
KeaP1	Kelch-like ECH-associated protein 1
LOOH	Lipid hydroperoxides
MDA	Malondialdehyde
MPC	Mitochondrial pyruvate carrier
NOX	NADPH oxidase
NLRP3	NOD-like receptor thermal protein domain associated protein 3
Nrf2	Nuclear factor erythroid 2-related factor 2
NF-κB	Nuclear factor kappa-B
PRR	Pattern recognition receptors
PPARa	Perixisome proliferator-activated receptor alpha
PI3K	Phosphoinositide 3-kinase
PUFA-PE	Polyunsaturated fatty acid-phosphatidyl ethanolamine
PUFA	Polyunsaturated fatty acid
PKA	Protein kinase A
Akt	Protein kinase B
ROS	Reactive oxygen species
RAGE	Receptor for AGE
RAASi	Renin–angiotensin–aldosterone system inhibitor
STAT	Signal transducer and activator of transcription
SIRT1	Sirtuin 1
SGLT2i	Sodium-dependent glucose transporters 2 inhibitor
SREBP-1c	Sterol regulatory element binding protein-1c
SOD	Superoxide dismutase
TFR	Transferrin receptor
TG	Triglycerides
ACTN4	α-actinin-4
α-SMA	α-smooth muscle actin
